# FOXO3 Is Expressed in Ovarian Tissues and Acts as an Apoptosis Initiator in Granulosa Cells of Chickens

**DOI:** 10.1155/2019/6902906

**Published:** 2019-07-11

**Authors:** Can Cui, Shunshun Han, Huadong Yin, Bin Luo, Xiaoxu Shen, Fuling Yang, Zihao Liu, Qing Zhu, Diyan Li, Yan Wang

**Affiliations:** Farm Animal Genetic Resources Exploration and Innovation Key Laboratory of Sichuan Province, Sichuan Agricultural University, Chengdu, Sichuan 611130, China

## Abstract

*FOXO3*, which encodes the transcription factor forkhead box O-3 (FoxO3), is a member of the FOXO subfamily of the forkhead box (FOX) family. FOXO3 can be negatively regulated by its phosphorylation by the PI3K/Akt signaling pathway and ultimately drives apoptosis when activated. In mammalian ovaries, the FOXO3 protein regulates atresia and follicle growth by promoting apoptosis of ovarian granulosa cells. Nonetheless, the specific effects of the FOXO3 protein on granulosa apoptosis of avian ovaries have not been elucidated. Therefore, we studied* FOXO3* expression in follicles with different organization and at all hierarchical levels of chicken follicles. Via an immunofluorescence assay, the chicken follicular theca at all hierarchical levels were found to be strongly stained with an anti-FOXO3 antibody. In chicken primary ovarian granulosa cells, mRNA levels of proapoptotic factors BNIP3 and BCL2L11 decreased in the absence of FOXO3, and so did PARP-1 and cleaved caspase 3 protein levels. After treatment with a recombinant FOXO3 protein, PARP-1 and caspase 3 protein levels increased, along with mRNA levels of Bnip3 and BCL2L11 (significantly,* p*<0.05). In addition,* FOXO3* was downregulated in chicken granulosa cells when different estradiol or FSH concentrations were applied. In conclusion,* FOXO3* is expressed in chicken reproductive tissues, including follicles and ovarian granulosa cells, and promotes apoptosis of chicken ovarian granulosa cells.

## 1. Introduction

Chicken laying performance has prominent and direct economic effects on the poultry industry; thus, it is necessary to discover the genetic mechanisms underlying this performance. There is a hierarchy of chicken ovarian preovulatory follicles. These follicles are of various sizes at different levels of maturation. Based on the development from small to large sizes, prehierarchical follicles can be subdivided into small white follicles (SWFs), large white follicles (LWFs), and small yellow follicles (SYFs), whereas hierarchical follicles can be classified into stages F1, F2, F3, F4, and F5 from large to small sizes [1]. Folliculogenesis is regulated by numerous factors, including hormones and growth factors, and is accompanied by a multitude of changes in gene expression. Hierarchical follicles filled with 5–7 yolks incorporated from the plasma grow rapidly before ovulating [2]. At this anatomical site, FSH accelerates yolk nutrient deposition by stimulating follicles. Meanwhile, FSH can also stimulate poultry SYFs, promote mRNA expression of the P450 side chain lyase (P450scc), and upregulate progesterone secretion to influence the selection of dominant follicles [3]. In addition, estrogen is secreted by chicken ovary gonads, promotes the growth of follicles, and inhibits granulosa cell apoptosis. Most follicles degenerate before ovulation in most mammals, a process called atresia [4]. In poultry, follicle atresia is often associated with follicle granulosa cells.

“FOX,” which stands for “forkhead box,” proteins are a superfamily of transcription factors. Since a forkhead gene was discovered and characterized in* Drosophila *in 1989, other members of this family have also been reported in succession [5]. The forkhead box O (FOXO) subfamily includes FOXO3 (FOXO3a/FKHRL1, FKHR-like 1), FOXO4 (AFX, acute-lymphocytic-leukemia-1 fused gene from chromosome X), and FOXO6. The FOXO family proteins are involved in a variety of processes, such as longevity, stress resistance, apoptosis, cell proliferation, differentiation, and metabolism [6]. The upstream pathway of FOXO members is regulated by the phosphatidylinositol 3-kinase (PI3K)–Akt (PKB, protein kinase B) pathway, with FOXO phosphorylated at three sites: Thr^32^, Ser^253^, and Ser^315^ [7]. There are some target genes related to apoptosis in the pathway downstream of the FOXO subfamily proteins, and this subfamily participates in the regulation of target genes or interacting partners within the cell [8]. Although members of the* FOXO* subfamily have similar sequences (resulting in function redundancy) [9], null mutations in* FOXO1*,* FOXO3*, and* FOXO4* yield specific phenotypes in mice and show that each* FOXO* gene has unique physiological effects and a distinct function [10].* FOXO3* is located at a mammalian and human cancer-associated site [t (6;11) (q21; q23)] and is highly similar to other* FOXO* gene family members, according to cDNA library screening [11]. Under normal conditions, FOXO3 binds to the 14-3-3 protein when phosphorylated and stays in the cytoplasm, preventing transcription of some downstream genes [12]. Conversely, FOXO3 may relocate to the nucleus when activating (not phosphorylated) and upregulating downstream genes [11]. One study by Castrillon et al. [13] revealed global activation of primordial follicles in* FOXO3*^*-/-*^ mice. In recent years, a wide variety of studies have shown that* FOXO3 *expression is closely related to primordial and primary follicles in mammals [14]. Research indicates [15] that when* FOXO3* is upregulated, the reproductive cycle of rats is significantly longer than that of the control group. All these studies suggest that the FOXO3 protein can serve as a regulatory factor for inhibiting the overactivation of original follicles and increasing the follicle reserves in the ovary in order to extend the reproductive period of females. Nevertheless, there is no report on the expression and mechanism of FOXO3 activity in poultry follicles during their development.

The purpose of this study was to elucidate the mechanism of* FOXO3* expression in chicken ovarian hierarchical follicles and granulosa cells. We either knocked down* FOXO3* or added a recombinant FOXO3 protein to investigate the proapoptotic effect of FOXO3 and the impact of FOXO3 on the expression of FASLG (TNF superfamily, member 6), BCL2L11 (Bcl-2-like protein 11), Bnip3 (Bcl-2/adenovirus E1B 19 kDa-interacting protein 3), and CDKN1B (cyclin-dependent kinase inhibitor 1 B). Besides, the effects of FOXO3 on granulosa cell apoptosis were also assessed by treating granulosa cells with different concentrations of FSH or estradiol (E_2_).

## 2. Materials and Methods

### 2.1. Ethics Statement

The animal experiment was conducted with the permission of the Committee on Experimental Animal Management of Sichuan Agricultural University, permit number 2014-18, which was issued on the basis of the Regulations for the Administration Affairs Concerning Experimental Animals of the State Council of the People's Republic of China. All chickens involved in this study were euthanized as painlessly as possible.

### 2.2. Preparation of Chicken Ovarian Granulosa Cell Samples

Ovaries were collected from Luhua chickens on the experimental farm for poultry breeding at Sichuan Agricultural University (Ya'an, China). As previously reported [16], laying hens at 40–45 weeks of age were caged under conditions of 16 h:8 h light:dark photoperiod and had* ad libitum* access to feed. Hens were killed by cervical dislocation 4–8 h before the next oviposition. Then, the whole ovary was quickly excised and placed in warmed (37°C) PBS (Sigma), and prehierarchical and hierarchical follicles were separated from the ovaries. The granulosa cells were dissected from the follicle layer in accordance with another study [17] and dispersed in PBS (Sigma) [18]. Cell viability was evaluated by the Trypan blue method and was usually > 90%. The cells were resuspended in Medium 199 (M199) supplemented with 2 mmol/L glutamine (1×), 40 mmol/L sodium bicarbonate (1×), 1% (v/v) PSA (antibiotic–antimycotic solution) (Sigma, England), and 5% charcoal-stripped fetal bovine serum (FBS) (Gibco BRL, Paisley) as described by Onagbesan et al. [19], and granulosa cells were seeded at 0.1 × 10^5^ viable cells per well in 12-well plastic plates (Corning Inc., Corning). The cells were cultured for 24 h in a humidified atmosphere containing 5% CO_2_ at 37°C to establish cell cultures. All granulosa cells used in subsequent experiments were subjected to these conditions.

### 2.3. RNA Extraction, cDNA Synthesis, and Real-Time RT-PCR

Chicken reproductive tissues and follicles were obtained from five Luhua hens aged 300 days from the same batch of a local farm. Total-RNA samples from tissues, follicles, and granulosa cells were isolated using TRIzol Reagent (Invitrogen, Carlsbad, CA, USA) and then treated with DNase (Promega, Madison, WI, USA).

RNA quality and concentration were evaluated on an Agilent 2100 Bioanalyzer (Agilent Technologies, Santa Clara, CA, USA), and first-strand cDNA was synthesized from total RNA. Primers used for real-time RT-PCR are shown in Table 1. Real-time RT-PCR was carried out on a LightCycler system (Roche, Basel, Switzerland). All mRNA expression levels were normalized to *β*-actin mRNA level, and then the mean expression level in each group was computed.

### 2.4. Western Blotting

Protein samples from individual experiments were pooled for western blotting analysis. The following primary antibodies were employed: anti-FOXO3 (Abcam, San Francisco, CA, 1:500 dilution), anti-*β*-actin (N21, Santa Cruz Biotechnology, Dallas, TX, 1:1,000 dilution), anti-FLAG (Sigma, 1:500 dilution), anti-histone 3 (Abcam, 1:500 dilution); anti-PARP-1 (Sigma, 1:500 dilution); anti-caspase-3 (Zen Bio, 1:500 dilution), anti-ER*β* (Zen Bio, 1:1000 dilution), and anti-FSHR (Zen Bio, 1:1000 dilution). The Flag-FOXO3 protein, source species chicken, was purchased from LMAI-BIO (China). The membranes were incubated with antibodies at 4°C overnight and then washed in buffer (10 mM Tris-HCl, pH 7.5, 100 mM NaCl, and 0.1% [v/v] Tween 20). Next, the membranes were treated with a horseradish peroxidase-conjugated IgG antibody (Santa Cruz Biotechnology, 1:2,000 dilution) for 2 h at room temperature. Chemiluminescence was analyzed to obtain the results. The relative expression of proteins was analyzed using the Quantity One software.

### 2.5. Immunofluorescence Experiment

A paraformaldehyde solution (buffered) was used for fixing the theca of freshly isolated hierarchical follicles in a physiological pH range. Immunofluorescence staining was performed as previously described [20]. Tissues were briefly submerged in a 30% sucrose solution and then sectioned at 10 *μ*m thickness using a microtome at a freezing temperature (Leica CM1520). The sections were incubated with the anti-FOXO3 antibody (Novus, 1:100) for 1 h at room temperature and then incubated with an Alexa Fluor 555 (red)-conjugated goat anti-rabbit IgG antibody (Invitrogen, 1:250) for 1 h in the dark. After staining with 4′,6-diamidino-2-phenylindole (DAPI, Invitrogen) for 20 min, the sections were washed, mounted on slides, and observed under a laser-scanning confocal microscope.

### 2.6. Cell Transfections and Treatments

#### 2.6.1. Gene Knockdown

Small interfering RNA (siRNA) was transfected when the fusion degree of the chicken granulosa cells* in vitro* reached ~65%–70%, as seen under the microscope. The medium was replaced with a serum-free medium (Opti-MEM, Invitrogen), and blank and negative control groups were set up simultaneously. The cells were incubated with 0.5% (v/v) Lipofectamine 3000 (Invitrogen) and 0.3 mM FOXO3 siRNA each and then incubated for 6 h. After that, the medium in the 12-well plates with the transfected cells was replaced with a 1× complete medium, followed by incubation in a cell culture incubator for 36 h at 5% CO_2_ and 37°C.

#### 2.6.2. Recombinant FOXO3 Protein Treatment

After the granulosa cells were attached to the 6-well plate, the cells were washed twice with a serum-free medium (DMEM). The culture solution was removed when the cells reached 70% confluence, and the cells were further transfected with different concentrations (0, 50, 100, or 200 ng/mL without serum) of exogenous recombinant FOXO3-Flag (ProSpec, Israel) protein using the *μ*-Proteofection Kit (ibidi, Germany) as the transfection reagent. The cells were cultured for 24 h, at 2 ml of the medium per well, and each treatment had three replicates. Then, RNA was extracted to detect changes in the expression of the target genes.

#### 2.6.3. E_2_ or FSH Treatment

After the cells adhered to the 6-well plate, the granulosa cells were rinsed with PBS twice. Then, the cells were grown in a serum-free medium containing different concentrations of E_2_ (ProSpec, Israel) (0, 10, 20, or 50 *μ*mol/L) or FSH (ProSpec, Israel) (0, 5, 10, or 20 ng/mL) for 12 h. After that, the medium was replaced with the complete medium, and the cell culture was maintained for additional 24 h with three replicates for each treatment group.

## 3. Results

### 3.1. FOXO3 mRNA Is Expressed in Various Reproductive Tissues of Chickens

**Figure 1:**
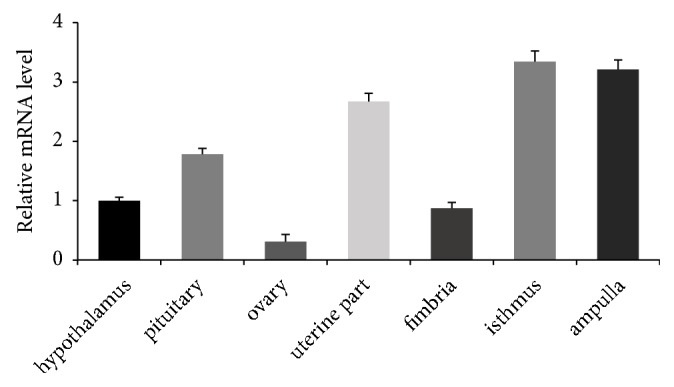
*FOXO3 mRNA is expressed in different chicken tissues*.* FOXO3* mRNA levels in various chicken tissues were examined by real-time RT-PCR. The measured* FOXO3* expression values were normalized to *β*-actin mRNA levels and are presented as a fold difference from hypothalamus values. Data are expressed as means ± SEM (n = 3 independent cell cultures).

*FOXO3 *mRNA levels in various tissues related to reproductive traits and granulosa cells of healthy chicken follicles are presented in Figure 1. Its expression in the tissues was not uniform, showing the highest expression in the ampulla and the lowest in the ovaries. In the fallopian tube, FOXO3 manifested the lowest expression in the umbrella region, followed by the uterine region, but it is obvious in the figure that FOXO3 expression in the uterus was significantly higher than that in the umbrella region and that the expression was higher in the isthmus than in the uterus.

**Figure 2:**
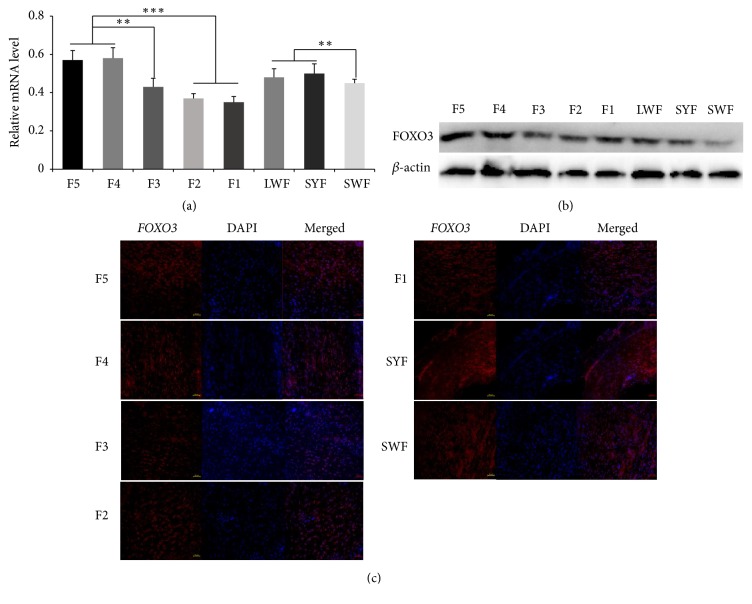
*FOXO3 mRNA and protein expression levels in chicken ovarian follicles*. (a) FOXO3 mRNA expression in chicken follicles at different developmental stages. (b) Western blot analysis showing FOXO3 protein expression in chicken ovarian follicles (*β*-actin served as a reference). (c) The FOXO3 protein was detected at all developmental levels of chicken follicular theca. Data are expressed as means ± SEM (n = 3 independent cell cultures). *∗*P<0.05; *∗∗*P<0.01, and *∗∗∗*P<0.001.

FOXO3 was expressed in follicles at all stages, and the expression level in follicles increased with increasing follicle hierarchy (Figure 2(a)). In hierarchical follicles, FOXO3 expression in F1 follicles reached the minimum, which was significantly lower than its expression in F2 and F3 follicles (*p *< 0.05) and strongly significantly lower than that in F4 and F5 follicles (*p *< 0.01). In prehierarchical follicles, FOXO3 expression in SYFs was significantly higher than that in SWFs (*p*<0.01), but not significantly different from that in LWFs (*p*>0.05).

### 3.2. FOXO3 Protein Is Expressed in Chicken Ovarian Follicles

The FOXO3 protein levels in chicken ovarian follicles at all stages were examined through western blotting. A representative photograph is presented in Figure 2(b), and *β*-actin served as a reference. The amount of the FOXO3 protein was higher in hierarchical follicles than in prehierarchical follicles.

### 3.3. FOXO3 Protein Is Expressed in the Chicken Follicular Theca

The localization of the FOXO3 protein in healthy chicken ovarian follicles was detected by an immunofluorescence assay (Figure 2(c)). The FOXO3 protein was successfully stained with the antibody at all follicle stages. Strong immunostaining was noted in SYFs on the surface of the follicle theca, whereas fluorescent staining was relatively weak in F1, F2, and F3 follicles.

### 3.4. Knockdown of FOXO3 Reduces Apoptosis in Chicken Granulosa Cells

**Figure 3:**
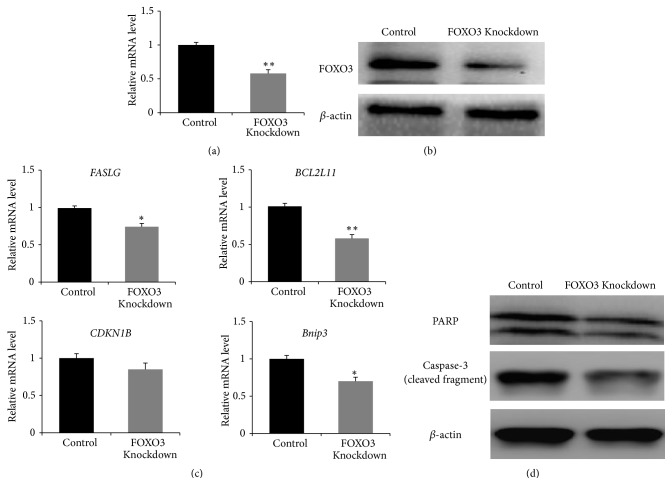
*FOXO3 knockdown in chicken granulosa cells*. (a) The relative mRNA expression level of* FOXO3* after the knockdown. (b) Western blotting analysis of FOXO3 protein levels after* FOXO3* knockdown. (c) The relative mRNA expression levels of the apoptosis-related genes after* FOXO3* knockdown. (d) Western blot analysis of cleaved PARP-1 and caspase 3 in control and FOXO3 knockdown cells. *β*-Actin served as a reference. Data are expressed as means ± SEM (n = 3 independent cell cultures). *∗*P<0.05; *∗∗*P<0.01.

To determine the efficiency of the designed FOXO3 siRNAs at knocking down the target gene expression, an experiment on chicken granulosa cells was carried out. After FOXO3 siRNA treatment, RT-PCR showed decreased expression of FOXO3 in these cells (Figures 3(a) and 3(b)). The mRNA levels of* FASLG*,* BCL2L11*, and* Bnip3*—the transcriptional targets of FOXO3 in mammal granulosa cells—remarkably decreased (*p*<0.05), while* CDKN1B* expression showed no significant change (*p*>0.05, Figure 3(c)). Furthermore, by western blotting, we analyzed cleaved caspase 3 and PARP-1, markers of the intrinsic apoptotic pathway, after FOXO3 knockdown in the cells. The results in Figure 3(d) indicate that PARP-1 and cleaved caspase 3 expression in chicken granulosa cells decreased relatively to the control. In general, these results revealed decreased apoptosis during FOXO3 knockdown in chicken granulosa cells.

### 3.5. Recombinant FOXO3 Protein Induces Apoptosis in Chicken Granulosa Cells

**Figure 4:**
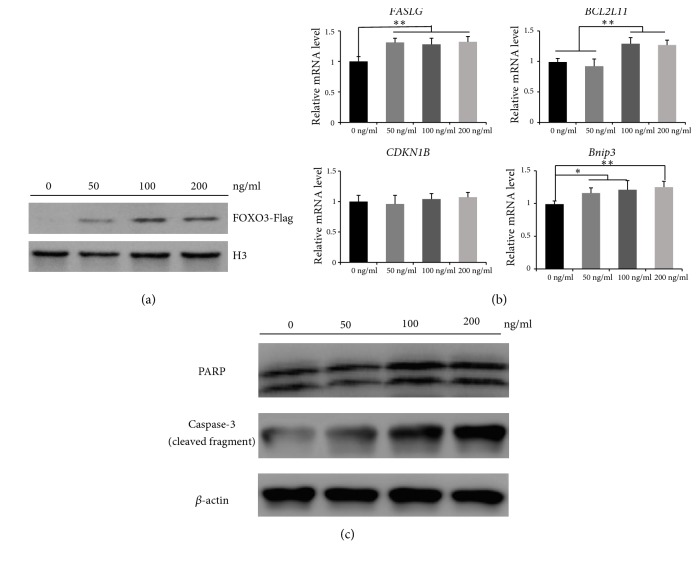
*Treatment of chicken granulosa cells with the exogenous recombinant FOXO3 protein*. (a) Western blot analysis of FOXO3 protein levels in extracted nuclear lysates; the relative expression level was normalized to that of H3. (b) The expression of apoptosis-related genes in chicken granulosa cells after treatment with different concentrations of the exogenous recombinant FOXO3 protein. (c) Western blot analysis showing the expression levels of PARP-1 and cleaved caspase 3 after recombinant-FOXO3 treatment. *β*-Actin served as a reference. Data are presented as means ± SEM (n = 3 independent cell cultures). *∗*P<0.05; *∗∗*P<0.01.

To explore the effects of the recombinant FOXO3-Flag protein on chicken granulosa cells, the FOXO3-Flag protein was added to the medium at different concentrations (0, 50, 100, or 200 ng/ml). To test whether the recombinant FOXO3 protein enters the nuclei of cells, we analyzed FOXO3-Flag protein levels in the nuclei and used H3 as a reference in western blots (Figure 4(a)). The results showed that the amount of the FOXO3-Flag protein in the nuclei increased in a dose-dependent manner. Meanwhile, mRNA expression levels of the key apoptosis genes,* FASLG*,* BCL2L11*, and* Bnip3*, increased significantly (*p*<0.05, Figure 4(b)). Next, we verified the protein levels of two apoptosis markers, cleaved caspase 3 and PARP-1, via western blotting.  Figure 4(c) illustrates increased PARP-1 and cleaved caspase 3 expression in a dose-dependent manner. Collectively, these data revealed increased apoptosis in the presence of abundant FOXO3.

### 3.6. E_2_ and FSH Each Reduces FOXO3 Expression in Chicken Granulosa Cell

**Figure 5:**
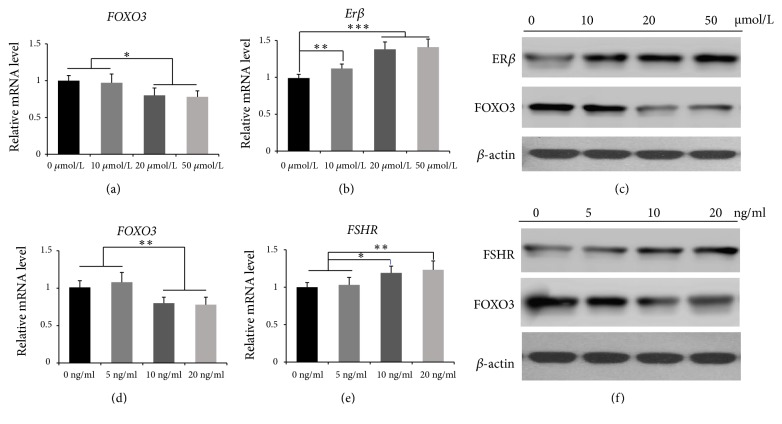
*E*_*2*_ *or FSH treatment of chicken granulosa cells*. (a) FOXO3 mRNA levels after treatment of chicken granulosa cells with different concentrations of E_2_. (b) ER*β* mRNA levels after treatment with E_2_ at different doses. (c) Western blot analysis showing ER*β* and FOXO3 expression levels after treatment with E_2_ (*β*-actin served as a reference). (d)* FOXO3* mRNA levels after treatment of chicken granulosa cells with different FSH concentrations. (e)* FSHR* mRNA levels after treatment with FSH at different doses. (f) Western blot analysis showing the amounts of FSHR and FOXO3 proteins after treatment with FSH (*β*-actin served as a reference). Data are expressed as means ± SEM (n = 3 independent cell cultures). *∗*P<0.05; *∗∗*P<0.01; *∗∗∗*P<0.001.

The chicken granulosa cells were treated with different concentrations of E_2_ (0, 10, 20, or 50 *μ*mol/L) or FSH (0, 5, 10, or 20 ng/ml). In the E_2_-treated group,* ERβ*, one of the genes controlled by the E_2_ receptor, was found to be upregulated as the concentration of E_2_ increased (Figure 5(a)), whereas FOXO3 expression decreased gradually (Figure 5(b), P<0.05). Western blotting showed the same results (Figure 5(c)). Similar results were obtained in the FSH-treated group:* FSHR* mRNA was found to be upregulated (P<0.05, Figure 5(d)), but* FOXO3 *mRNA was obviously downregulated by FSH treatment (P<0.05, Figure 5(e)). The same findings were made via western blotting (Figure 5(f)).

### 3.7. Discussion

In recent years, an increasing number of studies have demonstrated that members of the FOXO family play important roles in different hierarchical stages of follicular development. Park et al. [21] found that FOXO1 promoted apoptosis in mouse follicular granulosa cells. As one of the main members of the FOXO family, FOXO3 has mainly been studied in the ovaries of mammals, including humans [22], mice [23], pigs [24], and cattle [25], but it has not been studied in poultry.

Poultry follicles develop to various stages or undergo atresia through apoptosis at any stage of development. Thus, very few follicles can grow into dominant follicles and ovulate. Several studies [26] show that when FOXO3 acts on follicles, the PI3K/AKT signaling pathway is activated. AKT, also known as protein kinase B, is mainly regulated by the PI3K signaling pathway. FOXO3 can be phosphorylated by AKT and bind to the 14-3-3 protein in the cytoplasm, resulting in a temporary loss of its activity, that is, the inability to perform its function, thereby promoting follicular development in mammals. In our study, the results showed that the FOXO3 protein is expressed in the membranes of chicken follicles at different hierarchical stages. Subsequently, it was found that the FOXO3 protein expression level in follicles is similar to that of* FOXO3* mRNA. In chickens, expression of the* Bcl-2* gene, which inhibits apoptosis, reaches the maximum in F1 follicles, whereas almost no expression is detectable in LWFs [27]. These data are consistent with our finding that* FOXO3*, as a proapoptotic factor, is expressed most weakly in F1 follicles, suggesting that the FOXO3 protein may have a certain regulatory effect on chicken follicles. As early as 2003, studies [13] revealed that the mice with a* FOXO3* knockout show massive activation of original follicles, and this phenomenon mainly manifested itself via decreased gonadotropin levels. This finding implies that infertility is caused by the early use of original follicles. In subsequent studies, some researchers [28] have demonstrated that mice with* FOXO3* deletion are biologically younger than wild-type mice of the same age. Besides,* FOXO3-null* newborn mice show delayed follicular development, which increases the follicular reserve and improves fertility in adulthood. It can be inferred that downregulation of FOXO3 can maintain the functionality of the follicular reserve and improve fertility in mammals.

Follicular atresia caused by apoptosis of poultry granulosa cells is one of the main factors affecting the egg-laying efficiency in poultry [29]. The FOXO3 protein is a key factor in the INS/Igf-1 signaling pathway, and the expression and downregulation of FOXO3 regulate the expression of downstream target proapoptotic genes, such as* CDKN1B*,* FASLG*, and* BCL2L11*, whose products eventually lead to apoptosis [30]. In other studies, the activation of the transcription factor FOXO has been found to be critical for muscle atrophy [31]. Some autophagy-related genes, such as* LC3* and* Bnip3*, are regulated by the FOXO3 protein [32]. Overexpression of* FOXO3* in mouse nerve cells significantly promotes their apoptosis [33]. Likewise, overexpressed FOXO3 regulates apoptosis in testicular mesenchymal cells through BCL2L11 activity [34]. Studies have shown that, in follicle granulosa cells, upregulation of* FOXO3* can accelerate follicular atresia in pigs, indicating that the FOXO3 protein has a proapoptotic effect on mammalian granulosa cells [35]. On the one hand, via upregulation of CDKN1B, cell cycle proteins are inhibited, stopping the cell cycle at the G_0_–G_1_ transition, thereby blocking cell division. On the other hand, apoptosis is induced by the activation of proapoptotic cytokines BCL2L11 and FASLG [36]. In this study,* FOXO3* knockdown downregulated apoptosis markers (PARP-1 and cleaved caspase 3) and autophagy-related genes (*BCL2L11*,* Bnip3*, and* FASLG*). However, there was no significant change in CDKN1B expression; this phenomenon may be due to the suboptimal state of chicken cells* in vitro*. Meanwhile, following the treatment of chicken follicle granulosa cells with a high concentration of the recombinant FOXO3 protein, the resultant increase in PARP-1 and cleaved caspase 3 levels suggested that high concentrations of an exogenous recombinant FOXO3 protein may activate proapoptotic genes on the PI3K/Akt/mTOR pathway in chicken ovary granulosa cells. Collectively, these results mean that FOXO3 may act as an initiator of apoptosis in chicken granulosa cells, in agreement with the results from pigs [37].

One study [38] has revealed that FOXO1/3 knockout mice aged 25 days and 2 months showed granulosa cell–pituitary ovarian endocrine feedback loops, while the FSH level was barely detectable, suggesting a possible interaction between FSH and FOXO3. An earlier study [39] indicated that FSH promotes the proliferation of granulosa cells in rat ovaries, and our results are consistent with this conclusion.* FOXO3* expression levels significantly decrease with increasing FSH concentration, suggesting that FOXO3 may have a proapoptotic effect on chicken follicle granulosa cells, and its expression may be regulated by FSH. The latter can downregulate BimEL, a* FOXO3 *stimulator, and reduce apoptosis. Researchers have drawn a conclusion that the PI3K/Akt/FoxO axis may function downstream of FSH [40]. Nevertheless, in spite of the varied extent of* FOXO3 *downregulation at different FSH concentrations, the optimum FSH dose that can effectively inhibit* FOXO3* expression in chicken granulosa cells still requires further research.

The effects of E_2_ are mainly mediated by two estrogen receptors, ER*α* and ER*β* [41]. The proliferation of granulosa cells stimulated by E_2_ in mice is mediated by ER*β* [42]. Research has revealed that FOXO3 is actively degraded in ER-stimulated cells [43]. In this study,* FOXO3 *expression significantly diminished when the exogenously added E_2_ reached a certain concentration, in agreement with the above studies. Nevertheless, the specific mechanism of action of FOXO3—as a downstream effector of the PI3K/Akt cascade with a proapoptotic function—needs to be studied further.

In conclusion,* FOXO3* is expressed in chicken ovarian hierarchical follicles and granulosa cells and induces apoptosis, implying that FOXO3 may be a promising initiator of chicken follicular atresia.

## Figures and Tables

**Table 1:** Real-time RT-PCR primer sequences used in this study.

Gene	Primer sequence (5′-3′)
*FSHR*	F: GACAGAGATGTCCTTGGGTCT
	R: GCTCCCTTCGGAATGACTCT
*ER-β*	F: CCTGCTGGTATTGGCTCTCC
	R: ATAACACGCTTGGGCTCGAT
*BCL2L11*	F: ATCTCACTCGCTTGCAGAAG
	TGGCCCTCTTGAACTGAAAG
	TGGCCCTCTTGAACTGAAAG
	R: TTCCAGCACGGTTATCCAAG
*BNIP3*	F: AATGGGAATGGCAATGGAAAC
	R: TGTGAATGGAGATAGAAGCTGG
*CDKN1B*	F: CCGACTTCTACTTCAGGCAG
	R: GCAATTCCCGTTTACATCCAG
*FASLG*	F: GGAGAAGGAACTGGCTGAAC
	R: GGTTTCCTGTTAAGTGTGCTG
*FOXO3*	F: GTTTTGTAGCGTAGCCCCCT
	R: CACATTTTGGGGTGTGCCAG
*β*-Actin	F: GTCCACCGCAAATGCTTCTAA
	R: TGCGCATTTATGGGTTTTGTT

## Data Availability

The data used to support the findings of this study are available from the corresponding author upon request.
